# N-Glycosylation can selectively block or foster different receptor–ligand binding modes

**DOI:** 10.1038/s41598-021-84569-z

**Published:** 2021-03-04

**Authors:** Joni Vuorio, Jana Škerlová, Milan Fábry, Václav Veverka, Ilpo Vattulainen, Pavlína Řezáčová, Hector Martinez-Seara

**Affiliations:** 1 Department of Physics, University of Helsinki, P.O. Box 64, 00014 Helsinki, Finland; 2 Computational Physics Laboratory, Tampere University, PO Box 692, 33014 Tampere, Finland; 3 Institute of Molecular Genetics, Czech Academy of Sciences, Videnska 1083, 142 20 Prague 4, Czech Republic; 4 Institute of Organic Chemistry and Biochemistry, Czech Academy of Sciences, Flemingovo nam. 2, 166 10 Prague 6, Czech Republic; 5 Department of Cell Biology, Faculty of Science, Charles University, Vinicna 7, 128 00 Prague, Czech Republic; 6 MEMPHYS-Centre for Biomembrane Physics, Odense, Denmark

**Keywords:** Molecular modelling, NMR spectroscopy, Carbohydrates, Computational chemistry, Glycobiology, Post-translational modifications, Proteins

## Abstract

While DNA encodes protein structure, glycans provide a complementary layer of information to protein function. As a prime example of the significance of glycans, the ability of the cell surface receptor CD44 to bind its ligand, hyaluronan, is modulated by N-glycosylation. However, the details of this modulation remain unclear. Based on atomistic simulations and NMR, we provide evidence that CD44 has multiple distinct binding sites for hyaluronan, and that N-glycosylation modulates their respective roles. We find that non-glycosylated CD44 favors the canonical sub-micromolar binding site, while glycosylated CD44 binds hyaluronan with an entirely different micromolar binding site. Our findings show (for the first time) how glycosylation can alter receptor affinity by shielding specific regions of the host protein, thereby promoting weaker binding modes. The mechanism revealed in this work emphasizes the importance of glycosylation in protein function and poses a challenge for protein structure determination where glycosylation is usually neglected.

## Introduction

Glycosylation is a fundamental process where proteins are linked to complex oligosaccharides, glycans^1^. Most of the proteins at the extracellular side of eukaryotic cells contain covalently linked glycans^2^. Their structural roles include the mediation of interactions with the surrounding environment^3^, facilitation of correct folding^4–6^, and involvement in the assembly of membrane proteins^7^, also by direct interaction with lipids^8^. Glycans are also known to modulate the binding of ligands with several proteins, e.g., by masking the binding site^9–11^. Such regulation is relevant, especially in most immune processes, such as activation and homing, guided by regulated remodeling of the glycans^12^. However, the details of these modulation mechanisms are often poorly understood due to the glycans’ structural flexibility and dynamic nature^13,14^.

**Figure 1:**
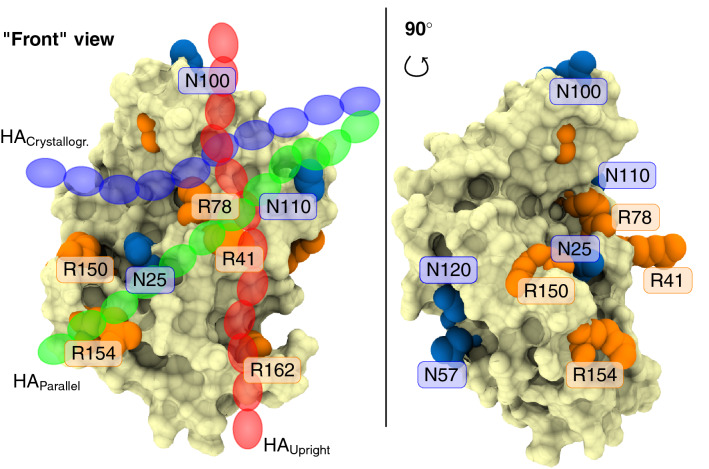
Human CD44-HABD (pale surface) with N-glycosylation sites (blue) and key HA binding arginines (orange) highlighted. Left panel also shows a schematic representation of HA in each binding mode (transparent blue, green, and red chains).

The transmembrane protein called CD44 is a key example of glycoproteins, whose functions are modulated by N-glycosylation^9,15–19^. Its primary task is to serve as a receptor for a carbohydrate polymer, hyaluronic acid (hyaluronan (HA))^20,21^. This ligand–protein interaction mediates a variety of physiological processes such as white blood cell homing, healing of injuries, embryonic development, and controlled cell death^22^. Recently, the CD44–HA interaction has also been utilized in the design of functional biomaterials^23^. CD44 binds HA exclusively via its lectin-like hyaluronate binding domain (HABD). In the canonical form, CD44 is a 722 residue-long type I transmembrane protein from which HABD comprises the first 150 amino acids (20-169) after the signal peptide^24,25^. Notably, human CD44-HABD contains five possible N-glycosylation sites (N25, N57, N100, N110, and N120)^24^ (see Fig. 1) that are known to be occupied by highly branched N-glycans, especially in various cancer cell lines^18,19,26^. The N-glycans elicit a dual effect on HA binding: while some glycan content favors the recognition of HA, the presence of negatively-charged sialic acids generally interferes or even blocks it^16,27,28^. However, the molecular mechanisms underlying such a dual effect remain unclear. In fact, most of the currently available structural data of HA–CD44 complexes are derived from non-glycosylated constructs^24,25,29,30^, leaving the structural details of fully N-glycosylated HABD elusive.

A shallow groove on the surface of the HABD forms the canonical binding site for HA. There the residue R41 stabilizes the binding in a pincer-like fashion^25,31,32^. In addition to this so-called crystallographic binding mode (blue chain in Fig. 1), in our previous work, we postulated the existence of two potential lower-affinity binding modes called parallel (green chain in Fig. 1) and upright (red chain in Fig. 1) modes^14^. These modes occupy the same general face of CD44-HABD, sharing to a large extent the R41-containing binding epitope. Additionally, each of these modes involves a second arginine residue that is distinct from that of the other binding poses^14^. As a result, each mode covers a unique region of the CD44-HABD surface. Such separation of the binding sites allows their selective silencing via antibodies that target different regions of CD44^33^. It also suggests that the presence of N-glycosylations may affect each of the binding modes differently. This idea is the central hypothesis of this work.

In this study, we employed atomistic molecular dynamics (MD) simulations to unravel how complex N-glycans at N25, N100, and N110 cooperatively cover the canonical binding groove of CD44-HABD. This sugar shield hinders the accessibility and ligand availability of the canonical binding groove significantly, thereby promoting the secondary upright HA–CD44 binding mode over the crystallographic binding site. We then used NMR complemented by atomistic MD simulations to show that a few short HA oligomers can bind CD44-HABD simultaneously at distinct binding sites. The observed binding sites correspond to the previously characterized crystallographic^25^, parallel, and upright binding modes^14^. We further reveal that anti-CD44 antibody MEM-85 does not cross-block the canonical HA binding site in non-glycosylated CD44. Instead, it blocks HA binding to glycosylated CD44^34,35^. These findings provide compelling evidence for the existence of a lower-affinity upright binding mode for HA. This binding mode overlaps with the binding site of MEM-85 and is promoted by N-glycosylation. The results demonstrate the existence of a new mechanism to control the ligand binding affinity of receptor proteins by promoting alternate binding sites by N-glycosylation.

## Results

### Complex N-glycans on CD44-HABD can cooperatively block its canonical binding site for hyaluronate

**Figure 2:**
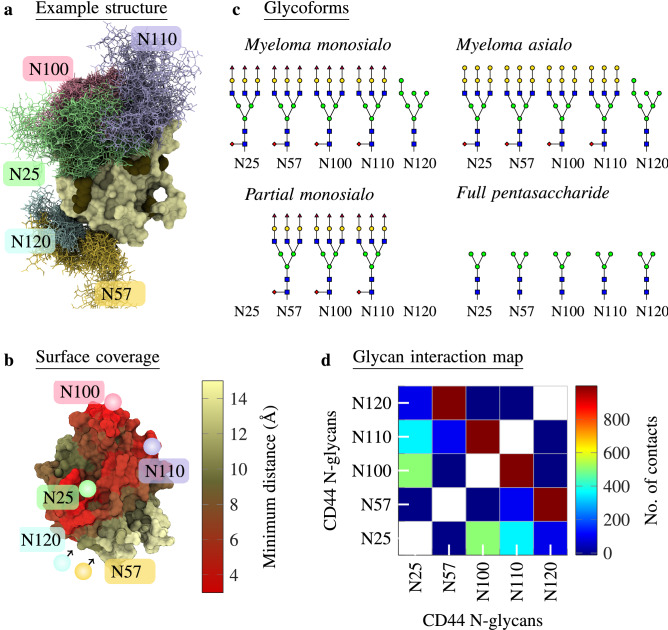
(**a**) Example structure of CD44-HABD with the *myeloma monosialo* glycoform (system G2 in Table 2). CD44-HABD is colored pale, and different colors separate the glycans. Glycans are depicted at every 50 ns in a trajectory of 1000 ns. (**b**) CD44-HABD with the surface colored according to the minimum distance to the N-glycans (not shown). Pale color corresponds to CD44–N-glycan distances over 15 Å, whereas bright red corresponds to distances less than 3 Å. The distance data have been averaged over 15 replicas (*myeloma monosialo* glycoform). (**c**) Glycoforms used in this study. The symbols follow the Symbol Nomenclature for Graphical Representations of Glycans^36^. (**d**) Number of contacts (defined as distance < 0.6 nm) between the five N-glycans on CD44-HABD (*myeloma monosialo* glycoform). The results have been averaged over time and 15 replicas. Another simulation force field (CHARMM36) in Notes SC and SD shows consistent data.

To characterize how N-glycans behave and fold on CD44-HABD, we in silico glycosylated a HABD structure (PDB:1UUH) with *myeloma asialo*, *myeloma monosialo*, *partial monosialo*, and *full pentasaccharide* N-glycan profiles (Systems G1–4 in Table 2 depicted in Fig. 2c). We then simulated each glycoform through 15 replicas. An average minimum distance between the complex N-glycans and the protein, as mapped onto the surface of HABD (Fig. 2b), reveals that in the *myeloma* glycoforms, the N-glycans cover a significant fraction of the protein surface. That is, with the complex oligosaccharides in *myeloma monosialo* and *myeloma asialo* glycoforms, the N25 glycan can interact intimately with the nearby N100 and N110 glycans, forming a sugar shield that covers the canonical binding site of hyaluronate (Fig. 2a). Furthermore, the contact map for the five N-glycans in the *myeloma monosialo* glycoform (Fig. 2d) shows the glycans at N25, N100, and N110 to establish, on average, several hundred intermolecular contacts, which are possible only if the three N-glycans become interconnected in the region that resides over the crystallographic hyaluronate binding groove. These results clearly show how complex N-glycans, facilitated by inter-N-glycan interactions, shield a significant portion of the hyaluronate binding face of HABD.

**Figure 3:**
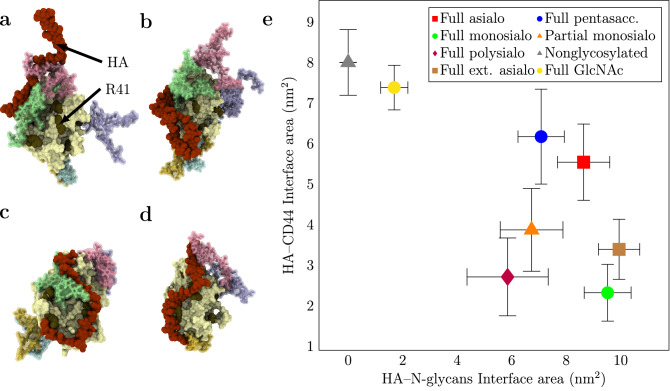
(**a–d**) Example structures ($t = 1000$ ns) of HA–CD44 complexes obtained in the spontaneous binding simulations (B3–6 in Table 2). The snapshots shown here are randomly selected from multiple simulation replicas. The color coding for HABD and N-glycans is the same as in Fig. 2. For comparison, examples of nonglycosylated CD44–HA complexes can be found from Ref. ^14^. (**a**) *full monosialo* glycoform (B4), (**c**) *full asialo* glycoform (B3), (**b**) *full polysialo* glycoform (B6), and (**d**) *partial monosialo* glycoform (B5). (**e**) Plot of HA–CD44 interface area vs HA–N-glycans interface area in different glycoforms. The data are calculated with GROMACS tool gmx sasa from systems B1–8. Error bars represent standard errors.

To study the spontaneous binding of HA to the N-glycosylated CD44-HABD, we performed simulations where both molecules were initially significantly separated. In this setting, the molecules can interact in a spontaneous manner without any apparent bias. These simulations refer to sets B1–8 in Table 2. Typical binding complexes arising from this set-up are shown in Fig. 3a–d.

Comparing the final ($t = 1000$ ns) HA–HABD and HA–N-glycans interface areas after the spontaneous binding of HA to the glycosylated HABD (Fig. 3e) reveals how the glycans hinder the recognition and how different glycoforms influence this process. While the HA–N-glycans interface obtains average values of 6–10 nm$^2$ with all glycoforms larger than *full GlcNAc*, the HA–CD44 interface varies significantly depending on the N-glycan content. The shortest *full GlcNAc* glycoform displays HA–CD44 binding similar to that of the non-glycosylated reference with recognizable binding modes, showing that these neutral sugar units do not obstruct the ligand binding. Instead, they offer more binding surface for HA compared to non-glycosylated HABD. Medium-sized neutral glycans (i.e., *full pentasaccharide* and *full asialo*) display HA–HABD interfaces ($\sim$6 nm$^2$) slightly lower than the non-glycosylated reference ($\sim$8 nm$^2$). While these glycoforms also provide additional interaction sites for HA through the larger size of the N-glycans, they also cover the important binding residues, preventing the formation of clear HA–CD44 binding modes. Agreeing with previous experimental findings, the sialylated glycoforms (*full monosialo*, *partial monosialo*, and *full polysialo*) display relatively low HA–HABD interfaces, with the partially glycosylated form showing the strongest binding to the protein. Together these results indicate that both the size of the N-glycans and charge (number of sialic acids) abrogate the binding of HA.

### N-glycans foster the occupancy of a secondary hyaluronate–CD44 binding mode

Table 1 lists the coverage of each of the three binding sites by the N-glycans. In the tested glycoforms (systems G1–4 in Table 2), amino acid residues distinct to the CD44-HABD binding modes exhibit a coverage of about 20 to 50%. The somewhat high standard errors indicate a large replica-to-replica variance in the folding of the N-glycans, as well as slow interconversion between the folding patterns. The use of 15 replicas, however, ensures a reasonable sampling of the possible patterns. In all cases, the crystallographic binding site is most significantly obstructed by the N-glycans, while the upright site is obstructed the least. Furthermore, coverage values calculated for the key hyaluronate binding residues of CD44-HABD (see Note SE) reveal how the key residues that are specific to the upright mode, such as K38 and R162, are generally less covered by the N-glycans. These observations imply that the lower-affinity upright mode is the most accessible binding configuration in a glycosylated CD44-HABD.

**Table 1:** Total N-glycan coverage of the residues involved in each binding mode. Data are calculated from systems G1–4 in Table 2. The contributions of each residue to each binding mode are extracted from our previous work^14^. The results indicate how much of the CD44-HABD surface (that is critical to hyaluronate binding) is covered by N-glycans.

Binding mode	Realistic asialo coverage (%)	Realistic monosialo coverage (%)	Partial monosialo coverage (%)	Pentasacharides coverage (%)
Cryst.	$\text {50} \pm \text {13}$	$\text {51} \pm \text {8}$	$\text {35} \pm \text {18}$	$\text {32} \pm \text {7}$
Parallel	$\text {48} \pm \text {14}$	$\text {46} \pm \text {11}$	$\text {34} \pm \text {17}$	$\text {26} \pm \text {6}$
Upright	$\text {29} \pm \text {12}$	$\text {30} \pm \text {7}$	$\text {21} \pm \text {13}$	$\text {16} \pm \text {4}$

**Table 2:** List of simulated systems.

System	Glycoform	HA	FF	Length (ns)	DOI data
G1$^{\rm{a}}$	Myeloma asialo	None	GLYCAM06	$\text {3}\times \text {5}\times \text {1000}$	10.5281/zenodo.3742147
G2$^{\rm{a}}$	Myeloma monosialo	None	GLYCAM06	$\text {3}\times \text {5}\times \text {1000}$	10.5281/zenodo.3742149
G3$^{\rm{a,b}}$	Partial monosialo	None	GLYCAM06	$\text {3}\times \text {5}\times \text {1000}$	10.5281/zenodo.3742154
G4	Full pentasaccharide	None	GLYCAM06	$\text {3}\times \text {5}\times \text {1000}$	10.5281/zenodo.3742158
B1$^{\rm{e}}$	Non-glycosylated	$\hbox {HA}_{{18}}$	GLYCAM06	$8 \times 1000$	10.5281/zenodo.4005682
B2$^{\rm{f}}$	Full GlcNAc	$\hbox {HA}_{{18}}$	GLYCAM06	$8 \times 1000$	10.5281/zenodo.4005689
B3$^{\rm{c,d,g}}$	Full asialo	$\hbox {HA}_{{18}}$	GLYCAM06	$8 \times 1000$	10.5281/zenodo.4005691
B4$^{\rm{d,f}}$	Full monosialo	$\hbox {HA}_{{18}}$	GLYCAM06	$8 \times 1000$	10.5281/zenodo.4005695
B5$^{\rm{b}}$	Partial monosialo	$\hbox {HA}_{{18}}$	GLYCAM06	$8 \times 1000$	10.5281/zenodo.4005701
B6$^{\rm{f}}$	Full polysialo	$\hbox {HA}_{{18}}$	GLYCAM06	$8 \times 1000$	10.5281/zenodo.4005707
B7	Full extended asialo	$\hbox {HA}_{{18}}$	GLYCAM06	$8 \times 1000$	10.5281/zenodo.4005740
B8	Full pentasaccharide	$\hbox {HA}_{{18}}$	GLYCAM06	$8 \times 1000$	10.5281/zenodo.4005743
C1$^{\rm{a,c,d}}$	Myeloma asialo	$\hbox {HA}_{{18}}$	CHARMM36	$\text {3} \times \text {1000}$	10.5281/zenodo.3742175
C2$^{\rm{a,c,d}}$	Myeloma monosialo	$\hbox {HA}_{{18}}$	CHARMM36	$\text {3} \times \text {1000}$	10.5281/zenodo.3742177
G5	Non-glycosylated	$\hbox {HA}_{{6}}$	GLYCAM06	$\text {20} \times \text {1000}$	10.5281/zenodo.3742160
G6	Non-glycosylated	$\hbox {HA}_{{6}}$	GLYCAM06	$\text {10} \times \text {1000}$	10.5281/zenodo.3742167

*Glycoform* tells the N-glycan content on the CD44 HABD in each system. *HA* tells whether HA was present and which kind. *FF* lists the simulation force field. *Length* lists the duration of the simulations. Glycoforms of CD44 were experimentally found in: $$^{\rm{a}}$$Ref.^26^, $$^{\rm{b}}$$Ref.^19^, $$^{\rm{c}}$$Ref.^9^, $$^{\rm{d}}$$Ref.^18^, $$^{\rm{e}}$$Ref.^24^, $$^{\rm{f}}$$Ref.^16^, and $$^{\rm{g}}$$Ref.^17^.

Strikingly, we observe minimal differences between the *myeloma monosialo* and *myeloma asialo* glycoforms, where the oligosaccharides are of the same length. However, the coverage values decrease notably with reduced glycan content in the *partial monosialo* or the shorter *full pentasaccharide* glycoforms. Like the myeloma-derived CD44-HABDs, the *partial monosialo* glycoform also displays a large number of contacts between the glycans N100 and N110 (Note SD). Their interaction is, however, less prone to disturb the crystallographic binding site as the N25-linked glycan is missing (Note SD). The *full pentasaccharide* glycoform is fully glycosylated but entails shorter oligosaccharides, which therefore limit the degree of protein coverage. These observations suggest that it is predominantly the degree of glycosylation and the size of the attached oligosaccharides that determine the coverage of the binding site. The inclusion of sialic acids has little effect on the coverage when compared to similar-sized non-sialylated N-glycans.

Figure S8 in Note SH compiled from the spontaneous binding simulations shows that the *non-glycosylated* HABD expresses the most interactions between HA and the arginines at the crystallographic binding groove (R41 and R78) compared to all the glycosylated HABDs. This indicates that the presence of N-glycans generally decreases the accessibility of these key HA binding residues. Consistently, the flanking arginines (R150, R154, and R162) are relatively more prone to interact with the ligand in the glycosylated cases, further suggesting that the binding modes involving these flanking arginines are activated in the glycosylated receptor. For additional observations from the spontaneous binding simulations, see Note SI.

### Antibody MEM-85 does not cross-block hyaluronate binding to non-glycosylated CD44

**Figure 4:**
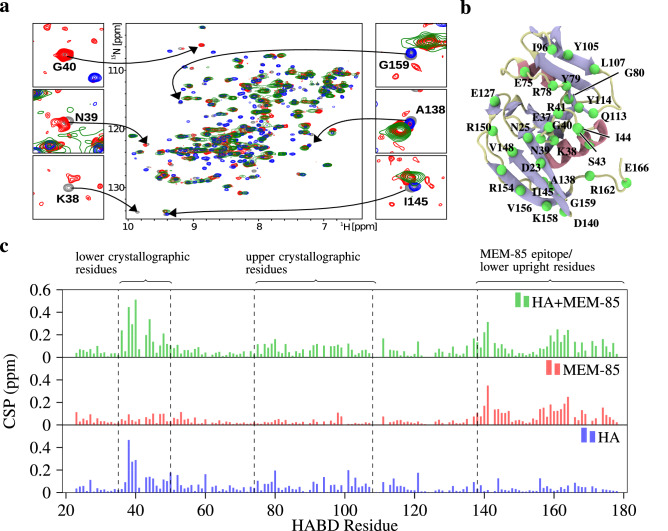
NMR confirms the simultaneous binding of scFv MEM-85 and hyaluronate to CD44-HABD. (**a**) 2D $^{\text {15}}$N/$^{\text {1}}$H HSQC spectra are shown for free $^{\text {15}}$N-CD44-HABD (grey), $^{\text {15}}$N-CD44-HABD in complexes with hyaluronate hexamer (threefold molar excess; blue), scFv MEM-85 (twofold molar excess; red), and both scFv MEM-85 (twofold molar excess) and hyaluronate hexamer (threefold molar excess; green). Details of the signals are shown for selected residues from the hyaluronate-perturbed (left) and antibody-perturbed (right) regions of CD44-HABD. (**b**) Illustration of the CD44-HABD (PDB:1UUH), highlighting the residues mentioned in the main text. From the residues, only the $\hbox {C}_{\upalpha }$ atom is depicted (green). Coloring of the protein is based on secondary structure, such that coils are pale, sheets are blue, and helices are red. (**c**) Histograms of the minimal combined chemical shift perturbation (CSP) versus the protein sequence are shown for $^{15}$N-CD44-HABD in complex with hyaluronate hexamer (blue), scFv MEM-85 (red), and both scFv MEM-85 and hyaluronate hexamer (green).

scFv MEM-85 antibody prevents the binding of hyaluronate to glycosylated CD44^34,35^. We used NMR to probe whether the same antibody prevents hyaluronate from binding a non-glycosylated CD44-HABD. The hyaluronate and antibody induced changes are clearly visualized in an overlay of the $^{\text {15}}$N/$^{\text {1}}$H HSQC spectra for the free $^{\text {15}}$N-CD44-HABD, $^{\text {15}}$N-CD44-HABD in complex with either hyaluronate hexamer (in a threefold molar excess), or scFv MEM-85 (in a twofold molar excess), and both scFv MEM-85 (in a twofold molar excess) and hyaluronate hexamer (in a threefold molar excess), see Fig. 4a. The observed changes can be interpreted as local perturbations/contacts in the vicinity of a given residue but they may also reflect a non-local secondary perturbation of some sort.

Residues from the hyaluronate-perturbed region such as K38, N39, and G40 exhibit similar spectral behaviour for the mixture of hyaluronate and antibody as for hyaluronate alone, i.e., their signals disappear. On the other hand, residues from the antibody-perturbed region^33^ such as A138, I145, and G159—necessary for upright mode—exhibit similar perturbations for the complex with both hyaluronate and the antibody, as in that of the antibody alone. Moreover, we calculated the histograms of the minimal combined chemical shift perturbation with respect to the free CD44-HABD spectra along its sequence (Fig. 4c). The obtained chemical shifts indicate that the spectra of the complex of CD44-HABD with both the antibody and hyaluronate still possesses the antibody-induced changes (residues within mainly the C-terminal segment of CD44-HABD) in addition to the hyaluronate-induced changes (residues within mainly the N-terminal segment of CD44-HABD). This clearly suggests the simultaneous binding of both hyaluronate hexamer and scFv MEM-85 to the non-glycosylated recombinant CD44-HABD.

In addition, the signals in the spectrum obtained for $^{15}$N-CD44-HABD in the presence of both antibody and hyaluronate hexamer are significantly broadened relatively to the signals in the spectra obtained for binary mixtures, as expected for a higher molecular weight of the ternary complex.

### Short hyaluronate oligomers bind to CD44-HABD simultaneously at distinct binding sites

**Figure 5:**
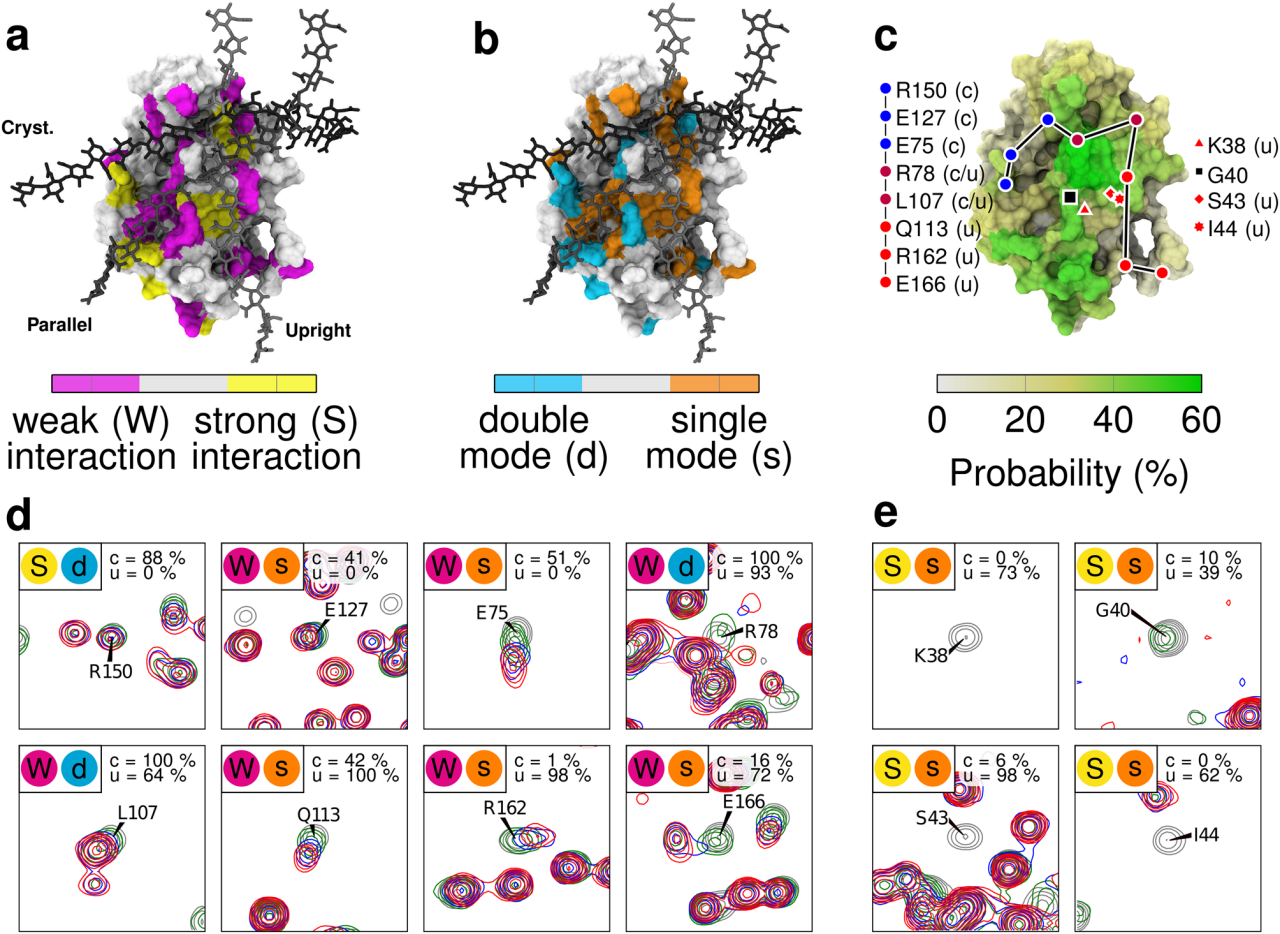
NMR titration of CD44-HABD by hyaluronate hexamer. Structures shown in a, b, and c are extracted from our previous study, where the CD44 coordinates are based on PDB:1UUH. (**a**) Selected $^{\text {15}}$N/$^{\text {1}}$H HSQC signals are sorted in two groups—residues with an immediate shift/disappearance of the signal, i.e., strong interaction (yellow color), and residues with a gradual shift, i.e., weak interaction (magenta color). (**b**) The signals are sorted in two groups — residues with a single signal, i.e., single binding mode (orange color), and residues with a doubled signal, i.e., double binding mode (cyan color). Hyaluronate hexadecamers are shown in three possible binding modes distinguished by shades of gray– the crystallographic *(cryst.)*, parallel *(parallel)*, and upright *(upright)* mode. (**c**) Hyaluronate-perturbed residues in simulations. The colored surface displays the probability of a given residue to be in contact with HA6 in our simulations (G5 in Table 2). Filled circles highlight the positions of selected residues from both the crystallographic and upright modes, which were perturbed by hyaluronate binding in our NMR experiments (cf. **d**). These marks are also colored based on the predominant binding mode of the highlighted residues in our earlier simulations^14^. Lines between the residues are drawn to guide the reader. The position of residues belonging to the R41-containing binding epitope is also shown (cf.**e**). Fig. S5 in Note SF shows similar surface data for both hexamer systems (Systems G5 and G6 in Table 2) (**d**). (**e**) Selected $^{\text {15}}$N/$^{\text {1}}$H HSQC signals are shown for free $^{\text {15}}$N-CD44-HABD (grey), $^{\text {15}}$N-CD44-HABD with equimolar hyaluronate hexamer (green) and twofold (blue) and a threefold (red) molar excess of hyaluronate hexamer. Colored circles (top left) indicate to which category the residue falls in (**a,b**). Probability of a given residue to interact with HA in each binding mode in our earlier simulations^14^ is indicated in the top right corner of each graph.

We analyzed the individual signals in the HSQC spectra for $^{15}$N-CD44-HABD titrated with hyaluronate hexamer; signals located in crowded areas of the spectra, including R41, were not taken into account to avoid ambiguity. This analysis revealed two trends (Fig. 5). Certain backbone amide group signals exhibited an instant shift or disappearance already at the hyaluronate to CD44-HABD ratio of 1:1, which indicates a strong interaction in the sub-$\upmu$M range of the respective residues with hyaluronate, while other signals shifted gradually during the individual titration steps, suggesting a relatively weaker interaction (>10 $\upmu$M) (Fig. 5a). In addition, several signals exhibited doubling, connected either with an instant shift or a gradual shift (Fig. 5b). This points out residues which interact only in a fraction of CD44-HABD molecules with hyaluronate, and/or interact in two different modes. Specifically, the signals of the following residues exhibited instant disappearance: K38, G40, G80, Y114; instants shift: S43, I44, Y79; instant shift with doubling: D140, R150, R154, V156, T174; gradual shift: D23, N25, E37, E75, I96, Y105, Q113, E127, V148, G159, R162, E166; and gradual shift with doubling: N39, R78, L107, K158, N164, D175.

Next, we mapped the critical residues involved in either strong or weak interaction with hyaluronate (Fig. 5c) and the residues interacting with hyaluronate in a single/double mode (Fig. 5d–e) onto the surface of a computational model of CD44-HABD (residues 20–169)^14^. This illustrates that the surface patches associated with all the three modes are affected in our hyaluronate titration experiments. Notably, the linear patch including residues K38, S43, I44, Y79, G80, Y105, Q113, Y114, R162, and E166 outlines the binding site for the upright binding mode. Moreover, the doubling of the signals in the C-terminal portion of CD44-HABD (residues D140, R150, R154, V156, K158, and N164) indicates the coexistence of the parallel and upright modes with the crystallographic mode. The NMR data, therefore, demonstrate that the short hyaluronate hexamer can, especially in higher molar excess, bind to non-glycosylated recombinant CD44-HABD simultaneously in several modes at distinct binding sites.

To further explore the simultaneous binding of hyaluronate on CD44-HABD, we performed a set of MD simulations with three hyaluronate hexamers binding to CD44-HABD (simulation G5 in Table 2). In these systems, the hyaluronate hexamers are initially in an unbound state (see Note SB), and thus, readily able to sample the space and find their respective binding sites during the course of the simulations (See Table S2 in Note SF). Fig. 5c shows the probability of the HABD surface to be in contact with HA, which correlates with the combined chemical shift perturbations observed in NMR. Additionally, Fig. S6 in Note SF shows a contact profile similar to the chemical shift profile recorded in NMR (Fig. 4), indicating that our experimental and computational results are in agreement.

## Discussion

We employed atomistic MD simulations and NMR to shed light on ligand–receptor interactions of CD44 and hyaluronate to unravel how N-glycosylation modulates the interactions. MD simulations showed that in the crystallographic mode (sub-$\upmu$M), N-glycans on CD44-HABD collectively shield the primary binding residues for hyaluronate. The shielding effect in this canonical binding mode is the strongest when complex type N-glycans occupy the N-glycosylation sites N25, N100, and N110. They are the most typical oligosaccharides found in these N-glycosylation sites^26^ and are sufficiently long to interlock over the canonical hyaluronate binding groove, thereby severely hindering its availability for the ligand.

Backing these observations, our HA binding simulations with glycosylated HABD show how the smaller N-glycan types, such as simple GlcNAc residue, lack both the reach and charge necessary to influence the binding of HA in a negative way. Instead, the presence of GlcNAc residues offers additional binding surface for HA, thereby possibly advocating the recognition of HA by providing additional polar interaction sites and minimal hindrance to the binding. This observation is in line with previous research that has shown with metabolic glycosidase enzymes that GlcNAc residues on CD44-HABD have a positive effect on the binding of HA^16^. Our simulations also show that once the size of the glycans on HABD increase, they start to prevent the entry of the ligand into its main binding site. High concentration of sialic acids further amplifies this effect through the increased size and negative charge of the glycans. Overall, our results from the spontaneous binding of HA to glycosylated HABD are in good qualitative agreement with previous experiments that have assessed the effect of different glycoforms, showing similar N-glycan-related size and charge-dependence for the binding of HA^9,16,27^.

We also found that the N-glycosylation of CD44-HABD promotes a secondary, less shielded but weaker (>10 $\upmu$M) hyaluronate binding site, which corresponds to the upright binding mode characterized previously by us^14^ and also suggested by others^24^. The results also revealed the degree of glycosylation and the size of the attached oligosaccharides to be the key factors in determining the coverage of the binding site, while the inclusion of single sialic acids to the glycan termini was found to have only a minor additional effect when glycans of equal length were compared to one another. Thus, it can be speculated, in the case of CD44–HA binding, that the binding-inhibiting role of monosialic acids stems from the more extended nature of the oligosaccharides and the resulting increase in the degree of coverage. The negative charge may play a more significant role in the case of polysialylated sugars, see Fig. 3e. Furthermore, if the glycosylation site N25 lacks sufficiently long glycans, the propensity to interlock with the N100 and N110 glycans decreases, thereby substantially decreasing the coverage of the crystallographic site, resulting in a more exposed site to the ligand. This is again in line with findings that have suggested some glycosylation patterns do not decrease the hyaluronate binding^16^.

Our NMR experiments support the notion of distinct hyaluronate binding sites on non-glycosylated CD44-HABD, which provides substantial evidence for the existence of separate hyaluronate binding modes. Strikingly, the residues perturbed in NMR match closely to those involved in the crystallographic, parallel, and upright binding modes. In our previous computational work, we illustrated the dynamic nature of the HABD–HA interactions outside the R41 epitope, especially in the case of the crystallographic binding mode (see Note SG). Similarly, the strong versus weak combined chemical shift perturbations in Fig. 5a show both the R41 epitope and upright groove to give predominantly strong interaction signals, while other regions flanking the R41 epitope tend to give out weak interaction signals, corresponding with the increased mobility of the bound HA in those regions. Despite the dynamic interactions, the importance of such weak binding sites to the overall strength of the binding is found to be high in a related protein–carbohydrate interaction^37^. The dynamics of the bound HA can be visualized in Note SG. Notably, while our NMR readouts support our computational findings, we cannot rule out the possibility of conformational changes as a reason for some of the observed perturbations.

The experimental results also agree well with the findings of our simulations of multiple hyaluronate hexamers with CD44-HABD, showing a similar hyaluronate–HABD binding profile (Fig. S6 in Note SF). The NMR readouts also show that the anti-CD44 antibody MEM-85 co-binds with hyaluronate on a non-glycosylated CD44, thus having a minimal effect on hyaluronate binding in this case. Conversely, the literature clearly states that MEM-85 blocks the hyaluronate binding of a glycosylated CD44^34,35^, implying the existence of a lower-affinity binding mode, whose binding site overlaps with the binding site of MEM-85. The MEM-85 epitope is known to be located around the residues Glu160, Tyr161, and Thr163^33^. As these residues are also a part of the upright mode, our results hint towards the existence of such binding.

Providing further evidence for the existence of the upright mode, when CD44-Ig (immunoglobulin) fusion proteins were expressed in COS cells and hence were presumably glycosylated, both MEM-85 and hyaluronate binding were significantly reduced by the mutation of K38 to arginine^34^. According to our previous work, K38 is exclusive to the upright mode^14^, which further implies that glycosylated CD44 favors to bind hyaluronate with the upright mode over the canonical crystallographic binding. We also note that distinct N-glycosylation profiles, e.g., ones that include an increased number of sialic acids, might cause different alterations to the binding.

CD44–HA interaction is known to display glycosylation-dependent levels of activation^9^ and binding affinities^16^. The activation levels have been attributed to varying degrees of sialylation^19,38^, yet the glycosylation dependent binding affinities could stem from the simultaneous masking of high-affinity binding sites and promotion of secondary sites. Such activation-dependent regulation of glycan remodeling is undoubtedly known to be a major mechanism driving cell motility, e.g., in the immune response^12^. CD44, in particular, is a hyaluronate-dependent leukocyte homing receptor that mediates both rolling interactions^39^ and cellular transmigration^40^. In such processes, tightly regulated affinity is required to enable dynamic velcro-like interactions between leukocytes and endothelial cells at inflamed tissue.

It is known that glycans stabilize or promote specific protein conformations^4–6^, dimer interfaces^3^, or orientations^8,41^, which ultimately affect ligand binding. There is also evidence of oligosaccharides that mask and shield specific parts of the protein surface^11,42^. N-glycosylations are also generally quite well known to protect large regions of the protein surface from, e.g., non-specific interactions or proteolytic cleavage^43^. The novelty of the present work lies in the fact that, in addition to all these features, N-glycosylation has an extremely valuable and hitherto unknown mechanism of action: N-glycosylation can control the affinity of ligand–receptor interaction by selectively blocking binding sites and promoting others.

## Methods

### Simulation system construction and models

We generated computational simulation models of glycosylated CD44-HABD. As the primary oligosaccharides, we employed fucosylated complex-type triantennary N-glycans, containing zero (asialo) or one (monosialo) terminal sialic acids per antenna, i.e., non-reducing termini. These oligosaccharide structures represent the predominant types in the so-called inducible (monosialo) hyaluronate binding phenotypes, together with a non-sialylated reference (asialo)^9,18,26^. To mimic the predominant CD44 glycovariants found recently in mouse myeloma cells^26^, we glycosylated N25, N57, N100, and N110 with the above-described complex type N-glycans and N120 with a triantennary high-mannose type structure without fucosylation (Fig. 2c). We call these glycoforms *myeloma asialo* and *myeloma monosialo*, depending on the degree of sialylation. Additionally, to emulate the mutant proteins lacking the N25 and N120 glycans that also lead to the inducible phenotype^19^, we constructed a monosialo glycoform, which lacks N-glycans at N25 and N120 (*partial monosialo*). Finally, we designed a fourth glycoform, where each of the five N-glycans is a charge-neutral core pentasaccharide (*full pentasaccharide*), to represent mildly glycosylated, less-cancerous cell types.

We constructed the simulation systems using the crystal structure of human CD44-HABD (PDB:1UUH^44^). We then followed the steps described in our previous work to curate the 1UUH structure^14^. This was followed by the in silico N-glycosylation of the HABD structure with the *doGlycans*^45^ tool. Before simulations, we inspected the ready-made glycan structures visually^46^ to confirm their correct configuration and stereochemistry. In every system, sodium and chloride ions were added to reach a typical physiological salt concentration of 150 mM, and to neutralize the charge of the system (Dang ions^47^ for AMBER99SB-ILDN and default ions for CHARMM36 systems). The systems were solvated with the recommended TIP3P water model^48^.

For each GLYCAM06-modeled system without a HA ligand (systems G1–4 in Table 2), we generated three different N-glycan starting configurations. Each configuration was used to start five replica simulations of 1000 ns, totalling to 15 replicas per glycoform. The CHARMM36 systems were simulated with three replicas (systems C1–2 in Table 2). Those three additional CHARMM36 systems had an added hyaluronate oligomer (18 monosaccharide units). We set them up initially to study HA binding to HABD, yet the oligomer never bound during the trajectories. Hence, we do not expect the hyaluronate to interfere with the folding of the N-glycans in those systems.

To understand how CD44 glycoprotein bind HA, we constructed GLYCAM06-modeled systems where $\hbox {HA}_{18}$ was let to spontaneously form a complex with different glycoforms of HABD (simulations B1–8 in Table 2). In the initial frame, we positioned the HA oligomer to the water phase, roughly 2.5 nm away from the R41 residue (the most important binding residue). The reasoning behind this initial distancing is to avoid any bias in the binding, see Note SB. In total, we studied seven different glycoforms: *full asialo*, *full monosialo*, *partial monosialo*, *full extended asialo*, *full pentasaccharide*, *full GlcNAc*, and *full polysialo*. These glycoform names are further explained in the Note SA. Lastly, we used *non-glycosylated* HABD from Ref.^14^ as reference system without glycosylation. For each glycoform, we performed eight replicas of 1000 ns, as listed in Table 2.

We also constructed an additional GLYCAM06-modeled system (20 replicas of 1000 ns) having non-glycosylated CD44-HABD together with three unbound (i.e., 1.5–2 nm from the protein surface as explained in Note SB) hyaluronate hexamers to study their spontaneous and simultaneous binding (system G5 in Table 2). That is, the carbohydrate fragments associated and/or dissociated from the protein spontaneously during the course of the simulation trajectories. Similarly, we generated systems (10 replicas of 1000 ns each) with CD44-HABD and two hyaluronate hexamers from which one was initially complexed to the crystallographic binding site, while the other was unbound (system G6 in Table 2). Tables S2 and S3 in Note SF list the observed association/dissociation cycles between the hexamers and HABD in simulations G5 and G6, showing that the sampling is adequate. All simulation data are publicly available in zenodo.org.

### Parameters for molecular dynamics simulations

Simulations were conducted using the GROMACS simulation software package^49^. For every simulation, we employed the following protocol. First, to relax clashes produced in the building process, we performed a short energy minimization run with the steepest descent algorithm (1000 steps). Subsequently, we performed 1 and 2 ns equilibration runs in the NVT and NpT ensembles, respectively, with coordinates of the protein and glycans restrained. Finally, we conducted production runs of different lengths (see Table 2).

The production runs, along with equilibration, employed the leap-frog integrator with a time step of 2 fs. During the runs, periodic boundary conditions were used in all three directions, and the LINCS algorithm was used to keep all bonds constrained^50^. Electrostatic interactions were treated with particle-mesh Ewald (PME)^51^ with a cut-off of 1.0 nm for the real part. Lennard–Jones interactions were cut off at 1 nm. Neighbour searching for long-range interactions was carried out every ten steps. The V-rescale^52^ thermostat was used to couple the systems to a heat bath of 310 K, while the Parrinello–Rahman^53^ barostat was employed to keep the pressure at 1 bar. At the beginning of each production simulation, we assigned random initial velocities using the Boltzmann distribution at the target temperature. The CHARMM36 simulations used the default parameters provided by CHARMM-GUI v1.7^54^. The simulation trajectories were saved every 100 ps. For other non-specified parameters, we refer to the GROMACS 4.6.7^55,56^ defaults for the AMBER99SB-ILDN/GLYCAM06 systems or to the GROMACS 5.1.4^49^ defaults for the CHARMM36 systems.

### Analysis of simulations

All distances and numbers of contact were calculated with gmx mindist tool from the GROMACS 5.1.4 package, using a cutoff of 0.3 nm unless stated otherwise.

N-glycan coverage for a given binding mode, $\textit{C}^{{\text {mode}}}$, is calculated by comparing the interactions of HA with a non-glycosylated HABD to the interactions of N-glycans with their core HABD. The calculation averaged over each replica is conducted as follows:

1 $$\begin{aligned} \textit{C}^{{\text {mode}}} = \left\langle \dfrac{\sum \limits _{{\text {res}}} \textit{C}^{{\text {mode}}}_{{\text {HA, res}}} \cdot \textit{C}^{{\text {rep}}}_{{\text {Glycan, res}}} }{\sum \limits _{{\text {res}}}{} \textit{C}^{{\text {mode}}}_{{\text {HA, res}}}}\right\rangle _{{\text {rep}}}. \end{aligned}$$

$\textit{C}^{{\text {mode}}}_{{\text {HA, res}}}$ is the average coverage of the protein residue “res” by HA in a given binding “mode”. The parameter $\textit{C}^{{\text {rep}}}_{{\text {Glycan, res}}}$ is the average coverage of the protein residue “res” by the N-glycans in each replica (“rep”). The average coverage ($\textit{C}_{{\text {res}}}$) is calculated as the ratio of frames where the distance between any atom of the HABD “res” to any atom of the target HA or N-glycans is closer than 0.3 nm.

### NMR spectroscopy

The proteins were expressed and purified as described in Ref.^33^. The $^{\text {15}}$N/$^{\text {1}}$H “heteronuclear single quantum coherence” (HSQC) spectra were acquired as described in Ref.^33^ using a 350 $\upmu$l sample containing 100 $\upmu$M $^{\text {15}}$N-labeled CD44-HABD, a 350 $\upmu$l sample containing 200 $\upmu$M $^{\text {15}}$N-labeled CD44-HABD and 210, 415, and 620 $\upmu$M hyaluronate hexamer (Contipro Group, Dolni Dobrouc, Czech Republic), using a 350 $\upmu$l sample containing 100 $\upmu$M $^{\text {15}}$N-labeled CD44-HABD and 200 $\upmu$M unlabeled scFv MEM-85, or using a 320 $\upmu$l sample containing 90 $\upmu$M $^{\text {15}}$N-labeled CD44-HABD, 180 $\upmu$M unlabeled scFv MEM-85, and 300 $\upmu$M hyaluronate hexamer. The sequence-specific resonance assignment for free CD44-HABD was obtained as published in Ref.^33^; signals could not be assigned for the following residues: Tyr42, Ser95, Asn100, Thr108, Ser109, Asn110, Ser112, Cys129 and prolines. The perturbations of $^{\text {15}}$N-labeled CD44-HABD signals in the HSQC spectra were monitored employing the minimal backbone chemical shift method ($^{\text {15}}$N and $^{\text {1}}$H)^57^.

## Supplementary Information

Supplementary Information.
