# Supplementary material for: N-Glycosylation can selectively block or foster different receptor–ligand binding modes
Source: Sci Rep. 2021 Mar 4;11:5239. doi: 10.1038/s41598-021-84569-z (PMC7933184; doi:10.1038/s41598-021-84569-z)
Supplement: Supplementary file 1 — Supplementary Information. [file 41598_2021_84569_MOESM1_ESM.pdf]

N-glycosylation can selectively block or foster different receptor–ligand binding modes — Supplementary Information

Joni Vuorio[1,2], Jana Škerlová[3,4], Milan Fábry[3], Václav Veverka[4,5], Ilpo Vattulainen[1,2,6], Pavlína Řezáčová[3,4], and Hector Martinez-Seara[4,*]

[1]Department of Physics, University of Helsinki, P.O. Box 64, FI-00014 Helsinki, Finland
[2]Computational Physics Laboratory, Tampere University, PO Box 692, FI-33014 Tampere, Finland
[3]Institute of Molecular Genetics of the Czech Academy of Sciences, Videnska 1083, Prague 4, 142 20, Czech Republic
[4]Institute of Organic Chemistry and Biochemistry of the Czech Academy of Sciences, Flemingovo nam. 2, Prague 6, 166 10, Czech Republic
[5]Department of Cell Biology, Faculty of Science, Charles University, Vinicna 7, 128 00 Prague, Czech Republic
[6]MEMPHYS - Centre for Biomembrane Physics
[*]hseara@gmail.com

Additional methods and results

SA Studied CD44 glycoforms

The N-glycan types used in this study are presented in Figure S1. Using these oligosaccharide types, we constructed several glycoforms of CD44 HABD, as listed in Table 2. The naming of each glycoform is composed of two parts (excluding the nonoglycosylated reference named as *non-glycosylated*): the first part signifies the degree of glycosylation and second part refers to the nature of the oligosaccharides used. In the first part of the name, 'full' refers to all the five N-glycosylation sites being occupied by N-glycan, while 'partial' means only sites N57, N100, and N110 are N-glycosylated. Term 'myeloma' refers to a glycoform where site N120 is glycosylated with high mannose type of oligosaccharides, while the remaining sites are N-glycosylated with the type of oligosaccharides given in the latter part of the name.

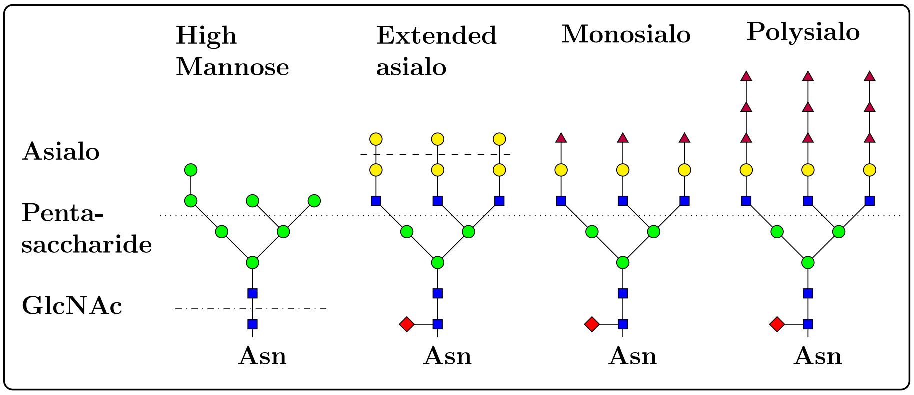

Figure S1. N-glycan structures employed for *in silico* glycosylation of the CD44 HABD. The horizontal dash dotted line separates the pentasaccharide core (below) from the antennae (above), while the short dashed line separates extended asialo N-glycan (above) from a shorter one (below). The dotted line separates the core pentasaccharide (below) from the antennae (above). Glycans are drawn according to the CFG symbol nomenclature.

The studied glycoforms represent a range of differently-sized CD44 glycoproteins, modeling the N-glycan structures

presumably present in the inactive (*full polysialo*), inducible (*full monosialo*, *partial monosialo*[1]), and active (*full asialo*, *full GlcNAc*) HA binding phenotypes, having varying sialic acid content and size[2,3]. *Full pentasaccharide* and *full extended asialo* glycoforms were generated to further evaluate the the effect of N-glycan size. With the *full monosialo* and *full extended asialo* glycoforms we were, for example, able to compare similar-sized glycans with and without sialic acids. The *myeloma* glycoforms mimic the predominant galactose-terminating and sialic acid-terminating CD44 glycovariants found recently in the mouse myeloma cells[4]. Non-glycosylated HABDs serve as a reference to the glycosylated proteins.

SB Avoiding simulation bias in the observed binding modes

When setting up any simulation system, one can bias the obtained results. To avoid favoring any particular binding mode between HA and HABD-CD44, the following two necessary steps were followed. First, we performed several replicas for each studied glycoform (Table 2). Second, HA was placed away from any potential binding site (1.5-2.5 nm). Here, we provide an example of such placing for the hyaluronate hexamers in simulations, see Fig. S2. This sort of placement reduces the risk of biasing HA's binding to any of the studied binding sites substantially. Simultaneously, the initial proximity enhances the chances of finding the binding modes in the allocated simulation time.

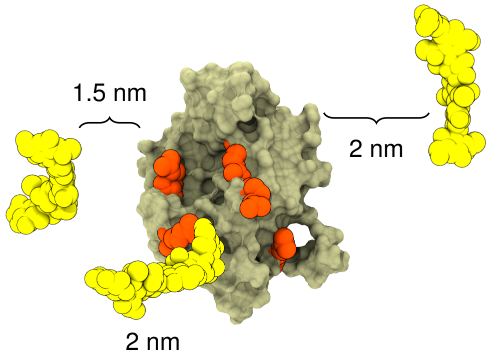

Figure S2. Representative starting configuration in System G5 simulations. Pale surface represents HABD; orange spheres are arginines R41, R78, R150, R154, and R162 (See Fig. 1; and yellow spheres are the HA hexamers. Each hexamer was placed randomly 1.5–2.0 nm away from the protein surface. This initial configuration quaranteed that the hexamers were able to sample the protein surface freely during the course of the simulations.

SC Force field comparison

All the critical simulations were repeated with the CHARMM36 force field (systems C1–2 in Table 2), and their conclusions were consistent with the combination of AMBER99SB-ILDN and GLYCAM06 discussed along the text. In this manner we can assure that the results are not force field-dependent.

Table S1 lists the coverage of each HA binding mode by the glycans using the CHARMM36 force field (simulations C1-2) in Table 2. These values are in general around 15 to 25 % smaller than that of the GLYCAM06 systems (see Table 1 in the main text). Yet, they also share the same trend: the crystallographic and parallel modes are the most and upright mode the least covered. In the CHARMM36 systems, the replica-to-replica variances are in general smaller than for GLYCAM06 as the N-glycans show greater mobility.

Table S1. Total N-glycan coverage of the residues involved in each binding mode using the CHARMM36 description. The results indicate how much of the CD44-HABD surface (that is critical to hyaluronate binding) is covered by N-glycans. For details of the analysis, see Methods above.

**Binding mode**	***Realistic asialo*: coverage (%)**	***Realistic monosialo*: coverage (%)**
Cryst.	38 ± 8	33 ± 7
Parallel	36 ± 7	36 ± 3
Upright	24 ± 4	20 ± 1

SD N-glycan versus N-glycan contacts

Figure S3 shows numbers of contacts between the five N-glycans on all tested glycoforms and force fields.

SE Interaction of hyaluronate binding residues with N-glycans

Figure S4 presents a contact histogram of the most prominent binding residues found from the literature[5]. It shows the binding of these residues to the N-glycans of each N-glycosylation site. For reference, the histogram also shows how these residues bind to hyaluronate oligomers (HA_{16} in our previous study[5]).

SF Binding profiles of hyaluronate hexamers in simulations

Figure S6 shows that three initially unbound HA_6 molecules bind to non-glycosylated CD44 HABD during 20×1000 ns trajectories. The occupied epitopes in HABD contacting the HA molecules are at residues 20–26, 38–42, 75–79, 87-100, 105–115, 141–161. These residues match the known epitopes for all three binding modes[5]. Qualitatively, we observe only one recognizable binding mode across 20 simulation replicas, with one HA fragment binding to the crystallographic binding mode. Yet, less distinct binding of the HA fragments occurred frequently at distinct binding sites. These observations along with the binding profile agree with the results of the NMR that show simultaneous binding of short HA fragments.

Figure S5 illustrates the probability of different HABD residues to be in contact with the HA hexamers durting the simulations. In panel **a**, the contacts are centered around the R41-centered binding epitope as well as residues in the extended region of HABD. This extended region is also known to undergo major conformational shift from ordered to disordered state upon the binding of ligand. In this region, e.g., arginine R154 have been noted to stabilize the ligand-bound conformation by interacting with the bound ligand in the disordered state of the receptor. In panel **b**), the high probability of contacts in the crystallographic binding groove is due to the other HA hexamer being initially placed into this position. The fact that there was zero dissociation events of the initially-bound HA hexamer clearly shows that the employed simulation force field is capable of reproducing the correct binding interaction.

Figure S6 also shows similar binding behavior for initially unbound HA_6 molecule that binds to HABD already occupied with another HA_6 in the crystallographic binding groove. The major binding epitopes locate roughly at residues 38–42, 105–115, 144–169. The C-terminal amino acids, 144–169, correspond to both the upright mode-specific HA binding residues and the residues that are influenced by MEM-85. This observation therefore agrees with the experimental observations and fortifies the notion of a second, lesser affinity binding site at the C-terminal portion of HABD.

Table S2. The number of association or dissociation events between individual HA hexamers and HABD during 1000 ns simulations. Data are calculated from simulation set G5 in Table 2 (i.e., HABD with three initially unbound HA hexamers, see Note SB). Association is counted when there are above 50 individual contacts between the atoms of CD44 and HA. Dissociation is counted when number of contacts reaches zero. Weak interactions (i.e., number of contact values between 0 and 50) are omitted.

**Replica**	1	2	3	4	5	6	7	8	9	10	11	12	13	14	15	16	17	18	19	20
$\mathbf{HA_6^1}$	11	1	1	1	1	9	1	1	6	1	3	7	5	7	3	1	3	5	3	7
$\mathbf{HA_6^2}$	1	1	3	1	1	5	7	3	4	1	1	2	1	1	1	1	1	1	1	5
$\mathbf{HA_6^3}$	1	3	11	3	7	2	8	1	5	3	1	1	1	1	3	1	5	9	1	3

Tables S2 and S3 show the number of attachments or detachments between each HA hexamers and CD44-HABD in simulation sets G5 and G6, respectively. One can see that each hexamer attaches to HABD at least once. However, many of the hexamers undergo multiple attachment-detachment cycles during the 1000 ns trajectories, with average number of attachments or detachments for the initially-free HA fragments being 3.1 ± 0.5. This indicates that the sampling of the binding surface is adequate. It also suggests that hexamers are most likely too short to readily form any of the possible binding modes at the studied time scales. Namely, hexamers are regarded as the minimum length of HA able to bind CD44[6]. Yet, the only hexamer that was placed into a predefined binding configuration in simulation set G6 remained in this configuration across all simulation repeats. This illustrates that the simulation force field is capable of reproducing the correct binding, thus giving further backing for the other results, too.

SG Example of CD44–hyaluronate binding

Figure S7 shows and example of CD44–hyaluronate binding in both the crystallographic and upright binding modes. As the ligand is depicted at various timestamps, it is easy to see which carbohydrate units interact the most with the protein.

Table S3. The number of association or dissociation events between individual HA hexamers and HABD during 1000 ns simulations. Data are calculated from simulation set G6 in Table 2. Association is counted when there are above 50 individual contacts between the atoms of CD44 and HA. Dissociation is counted when number of contacts reaches zero. Weak interactions (i.e., number of contact values between 0 and 50) are omitted. In these simulations, hexamers number 1 is attached to the crystallographic binding site, and no detachments are recorded. Hexamer number 2 is initially unbound similar to System G5. That is, it can readily sample the surface of HABD for possible binding sites.

**Replica**	1	2	3	4	5	6	7	8	9	10
$\mathbf{HA_6^1}$	0	0	0	0	0	0	0	0	0	0
$\mathbf{HA_6^2}$	5	5	3	4	4	1	0	3	3	2

SH Spontaneous binding of HA to glycosylated CD44-HABD

Figure S8 shows relative interaction probability of key arginine residues with HA in glycosylated receptors as compared to non-glycosylated CD44. The data show the flanking arginines R150, R154, and R162 to have relatively more interactions with the HA ligand in the glycosylated cases. The arginines R41 and R78 at the primary binding site, on the other hand, are less active in binding when the receptor is glycosylated. This illustrates how the sugar shield obstructs the primary binding site.

SI Additional observations from the spontaneous binding simulations

Highlighting the sialic acid-induced repulsive effect, HA failed to bind HABD entirely in one replica of the *full polysialo* systems. This is the only occasion with GLYCAM06 force field when we did not observe interactions between CD44 glycoprotein and HA during a 1000 ns trajectory.

The charge-neutral *full extended asialo* glycoform with two galactoses per antennae shows HA binding comparable to the equally-sized but charged *full monosialo* glycoform, which is again significantly lower than that of the shorter monogalactose-terminating *full asialo* glyform. This highlights the role of the size of the N-glycans in masking the binding site and thus determining the HA binding properties.

References

1. English, N. M., Lesley, J. F. & Hyman, R. Site-specific de-N-glycosylation of CD44 can activate hyaluronan binding, and CD44 activation states show distinct threshold densities for hyaluronan binding. *Cancer Res.* **58**, 3736–3742 (1998).

2. Lesley, J., English, N., Perschl, A., Gregoroff, J. & Hyman, R. Variant cell lines selected for alterations in the function of the hyaluronan receptor CD44 show differences in glycosylation. *The J. Exp. Medicine* **182**, 431–437, DOI: 10.1084/jem.182.2.431 (1995).

3. Zheng, Z., Cummings, R. D., Pummill, P. E. & Kincade, P. W. Growth as a solid tumor or reduced glucose concentrations in culture reversibly induce CD44-mediated hyaluronan recognition by Chinese hamster ovary cells. *J. Clin. Investig.* **100**, 1217, DOI: 10.1172/jci119635 (1997).

4. Han, H. *et al.* Comprehensive characterization of the N-glycosylation status of CD44s by use of multiple mass spectrometry-based techniques. *Anal. bioanalytical chemistry* **404**, 373–388, DOI: 10.1007/s00216-012-6167-4 (2012).

5. Vuorio, J., Vattulainen, I. & Martinez-Seara, H. Atomistic fingerprint of hyaluronan–CD44 binding. *PLoS computational biology* **13**, e1005663, DOI: 10.1371/journal.pcbi.1005663 (2017).

6. Teriete, P. *et al.* Structure of the regulatory hyaluronan binding domain in the inflammatory leukocyte homing receptor CD44. *Mol. Cell* **13**, 483–496, DOI: 10.1016/s1097-2765(04)00080-2 (2004).

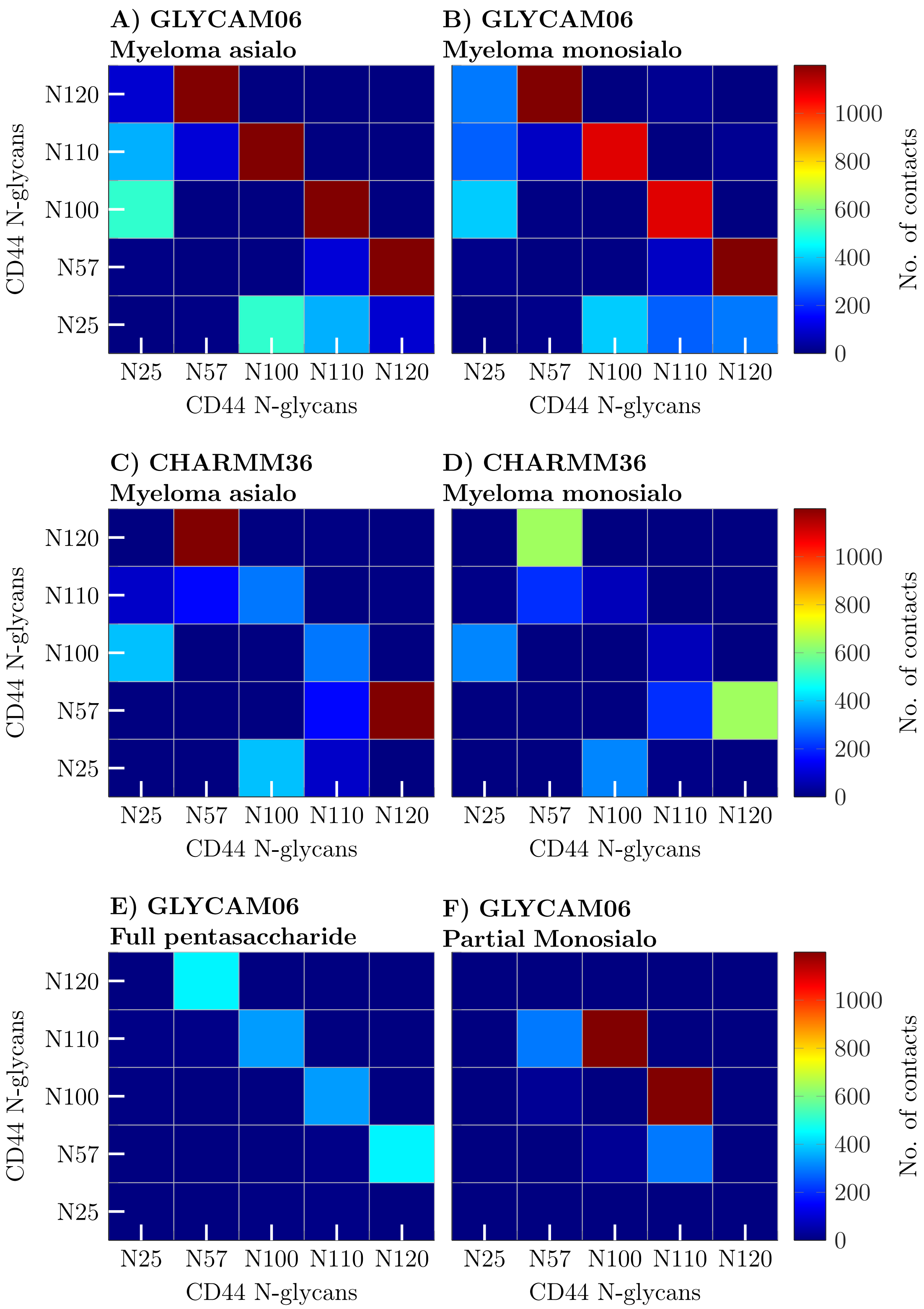

Figure S3. Number of contacts between the five N-glycans on CD44-HABD. Data is time and system averaged from 15 and 3 replicas for the GLYCAM06 and CHARMM36 systems, respectively. In the analysis, all the contacts were summed for each data point and a threshold for a contact was 0.6 nm.

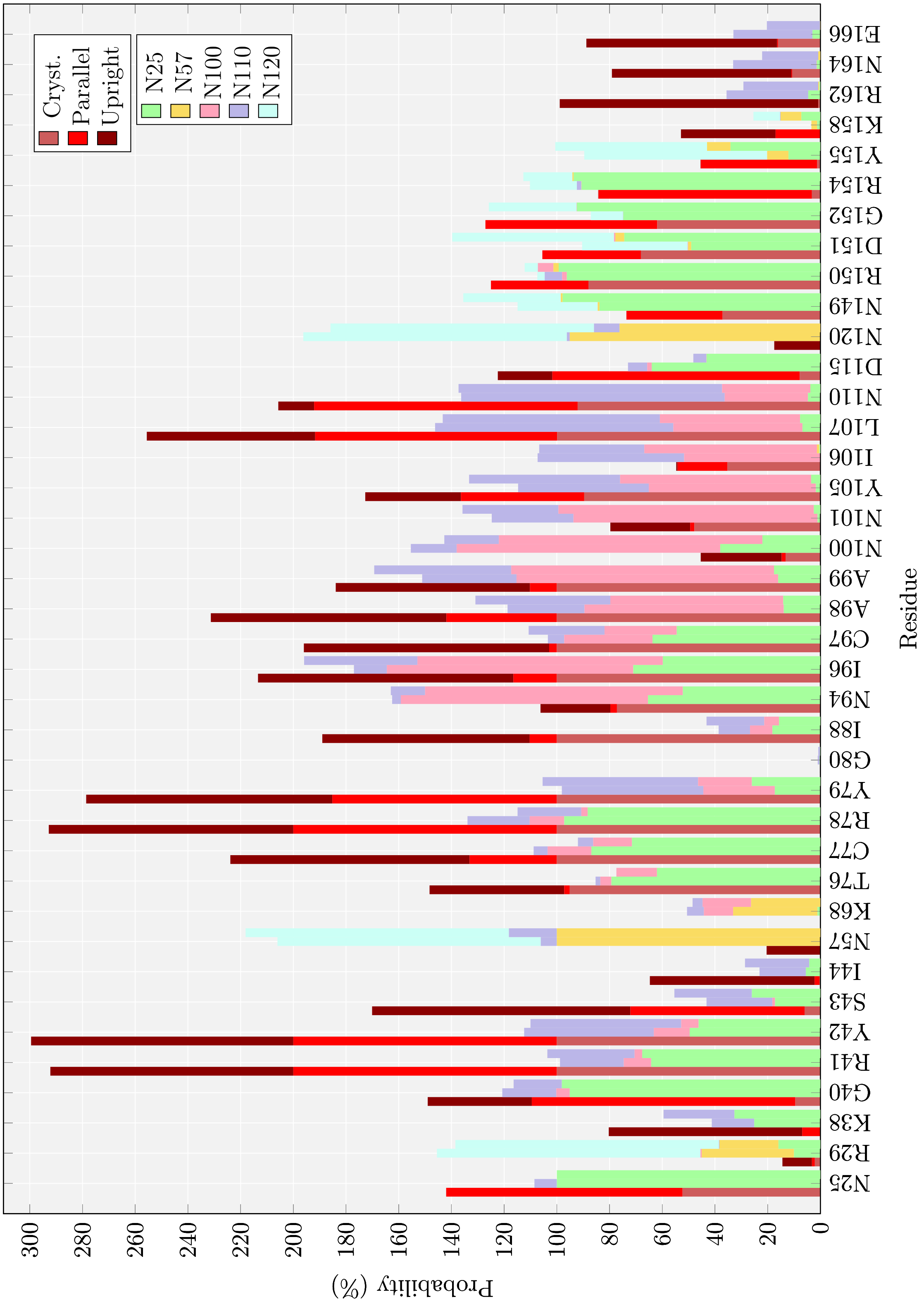

Figure S4. Contact histogram for the observed HA binding residues, according to the literature[5]. It shows in how many aggregate simulation frames (%) given binding residue–N-glycan interaction is present. These data are calculated from the *Myeloma monosialo* systems. For comparison, it shows in how many aggregate simulation frames (%) given binding residue–HA interaction is present for each binding mode (Data available in Ref[5]).

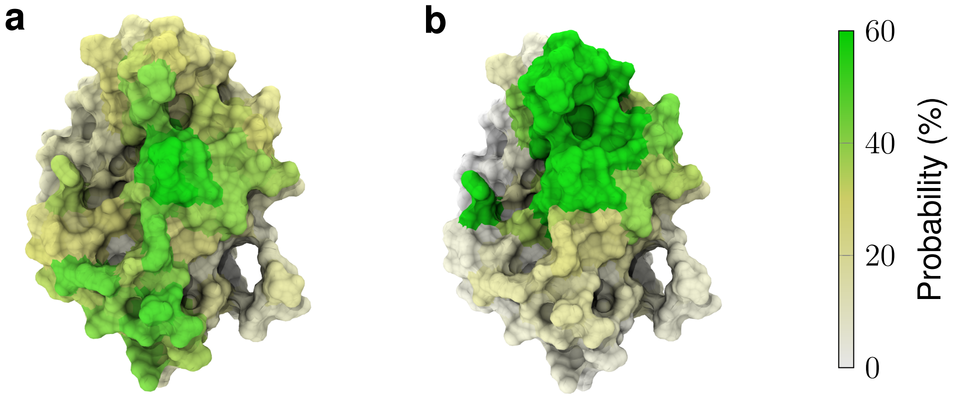

Figure S5. Hyaluronate-perturbed residues in simulations G5 (**a**) and G6 (**b**). The colored surface displays the probability of a given residue to be in contact with HA6 in our simulations. **a**: This simulation system entailed HABD and three randomly placed unbound HA_6 fragments. The observed binding was therefore spontaneous. **b**: This simulation system entailed HABD and one randomly placed unbound HA_6 fragment as well as one HA_6 fragment placed into the crystallographic binding mode. The observed binding of the unbound fragment was therefore spontaneous. Zero detachments of the bound fragment was detected during the simulations, see Table S3.

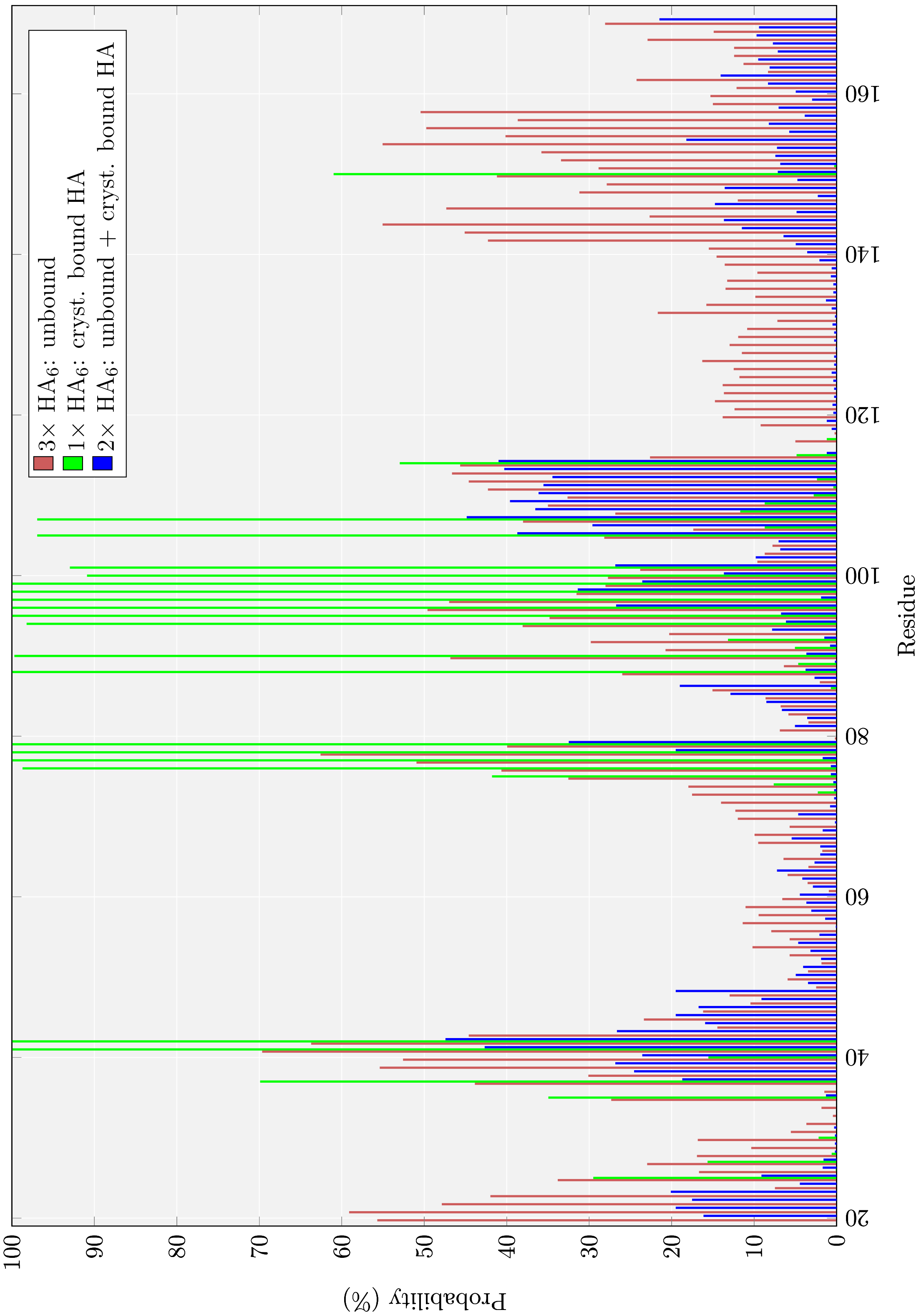

Figure S6. Contact histogram for all HA binding residues vs multiple HA_6. It shows in how many aggregate simulation frames (%) given binding residue–HA interaction is present. These data are calculated from the G5 and G6 systems in two settings: first, with either three initially unbound HA_6 molecules, or second, with two HA_6 molecules from which one is initially bound in the crystallographic mode and one is initially unbound, respectively. The data are calculated and averaged over 20 (system G5) and 10 (system G6) simulation replicas, see Methods for details.

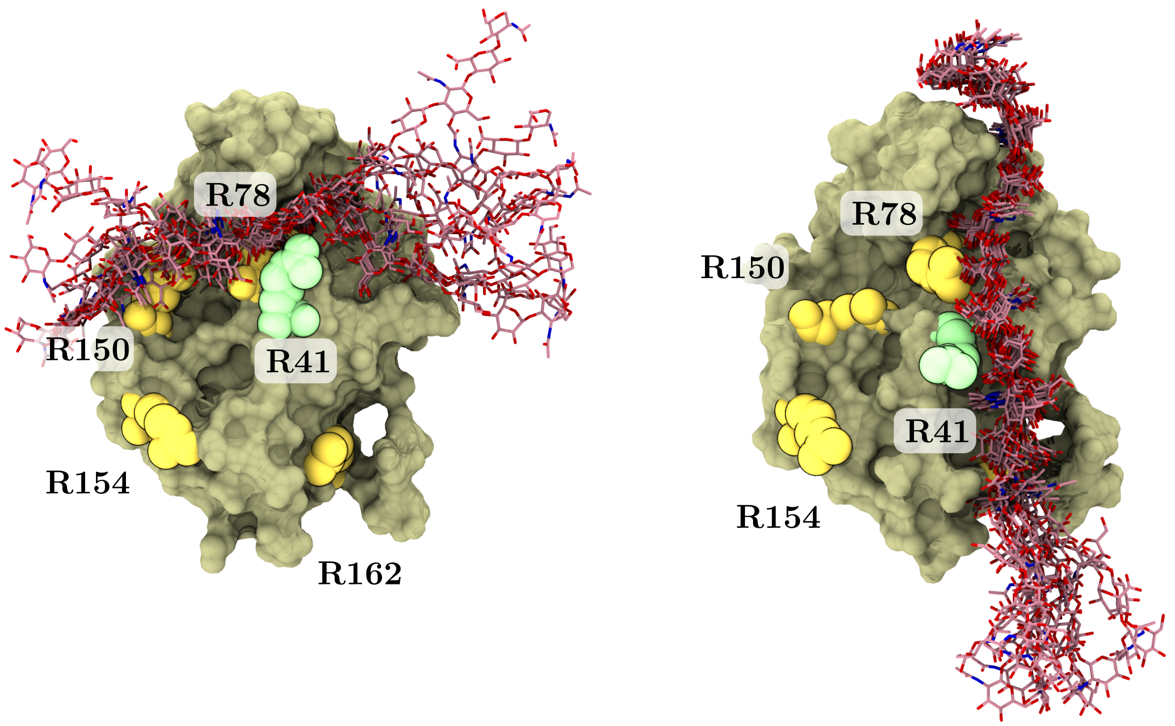

Figure S7. Dynamics of the HA ligand in crystallographic (left) and upright (right) binding modes. The tan surface depicts the protein surface in the first frame of a simulation. The red sticks represent the ligand drawn at every 50 ns into the simulation trajectory. R41 is colored light green. Other key arginines are colored yellow and labeled accordingly. Data are extracted from our previous simulations[5].

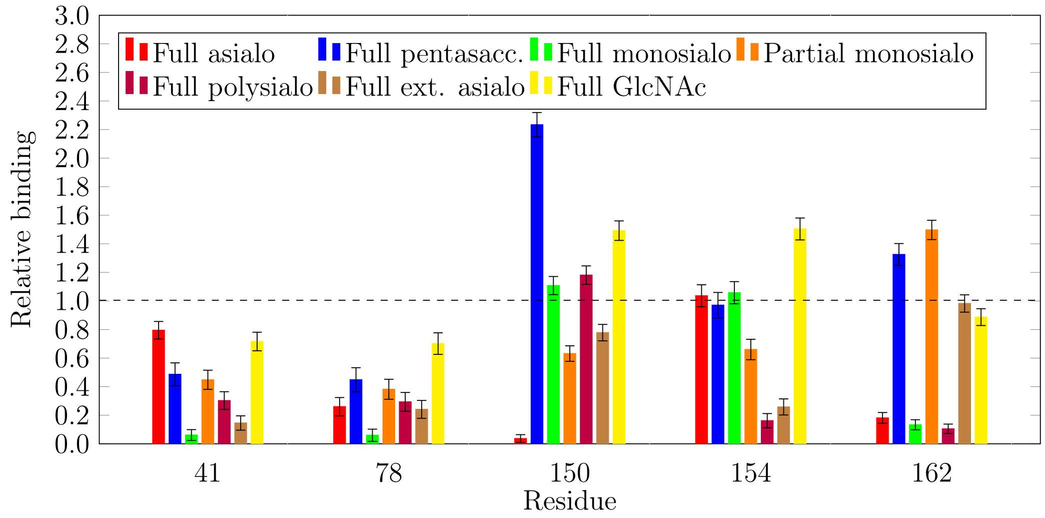

Figure S8. Relative interaction probability of HA with five CD44 arginines in different glycoforms. The data are calculated with GROMACS tool `gmx mindist` by counting the simulation frames in which a contact (HA–Arg minimum distance < 6 Å) is present (excluding the first 200 ns of binding). Values for each glycoforms are then normalized with the reference values from the non-glycosylated system in order to obtain the relative interaction probabilities. Values above 1 indicate an increase compared to the binding in the non-glycosylated case, while values below 1 indicate a decrease compared to the non-glycosylated case.
